# Patient and Practice Perspectives on Strategies for Controlling Blood Pressure, North Carolina, 2010–2012

**DOI:** 10.5888/pcd11.130157

**Published:** 2014-04-24

**Authors:** Katrina E. Donahue, Maihan B. Vu, Jacqueline R. Halladay, Cassandra Miller, Beverly A. Garcia, Doyle M. Cummings, Crystal W. Cene, Alan Hinderliter, Edwin Little, Marjorie Rachide, Darren DeWalt

**Affiliations:** Author Affiliations: Maihan B. Vu, Jacqueline R. Halladay Cassandra Miller, Beverly A. Garcia, Crystal W. Cene, Alan Hinderliter, Darren DeWalt, University of North Carolina, Chapel Hill, North Carolina; Doyle M. Cummings, East Carolina University, Greenville, North Carolina; Edwin Little, Marjorie Rachide, primary care practices in eastern North Carolina.

## Abstract

**Introduction:**

Patient and practice perspectives can inform development of team-based approaches to improving blood pressure control in primary care. We used a community-based participatory research approach to assess patient and practice perceptions regarding the value of team-based strategies for controlling blood pressure in a rural North Carolina population from 2010 through 2012.

**Methods:**

In-depth interviews were conducted with 41 adults with hypertension, purposely sampled to include diversity of sex, race, literacy, and blood pressure control, and with key office staff at 5 rural primary care practices in the southeastern US “stroke belt.” Interviews explored barriers to controlling blood pressure, the practice’s role in controlling blood pressure, and opinions on the use of team care delivery.

**Results:**

Patients reported that provider strategies to optimize blood pressure control should include regular visits, medication adjustment, side-effect discussion, and behavioral counseling. When discussing team-based approaches to hypertension care, patients valued verbal encouragement, calls from the doctor’s office, and the opportunity to ask questions. However, they voiced concerns about the effect of having too many people involved in their care. Practice staff focused on multiple, broad methods to control blood pressure including counseling, regular office visits, media to improve awareness, and support groups. An explicit focus of delivering care as teams was a newer concept.

**Conclusion:**

When developing a team approach to hypertension treatment, patients value high-quality communication and not losing their primary relationship with their provider. Practice staff members were open to a team-based approach but had limited knowledge of what such an approach would entail.

## MEDSCAPE CME

Medscape, LLC is pleased to provide online continuing medical education (CME) for this journal article, allowing clinicians the opportunity to earn CME credit.

This activity has been planned and implemented in accordance with the Essential Areas and policies of the Accreditation Council for Continuing Medical Education through the joint sponsorship of Medscape, LLC and Preventing Chronic Disease. Medscape, LLC is accredited by the ACCME to provide continuing medical education for physicians.

Medscape, LLC designates this Journal-based CME activity for a maximum of 1 **AMA PRA Category 1 Credit(s)™**. Physicians should claim only the credit commensurate with the extent of their participation in the activity.

All other clinicians completing this activity will be issued a certificate of participation. To participate in this journal CME activity: (1) review the learning objectives and author disclosures; (2) study the education content; (3) take the post-test with a 70% minimum passing score and complete the evaluation at www.medscape.org/journal/pcd (4) view/print certificate.

**Release date: April 24, 2014; Expiration date: April 24, 2015**

### Learning Objectives

Upon completion of this activity, participants will be able to:

- Distinguish significant variables identified by patients as barriers to better blood pressure control
- Distinguish significant variables identified by healthcare professionals as barriers to better blood pressure control
- Analyze what patients believe that healthcare professionals can do to help them improve their blood pressure control
- Evaluate attitudes of patients and healthcare professionals toward team-based care for hypertension

**EDITORS**

Rosemarie Perrin, Technical Writer/Editor, *Preventing Chronic Disease*. Disclosure: Rosemarie Perrin has disclosed no relevant financial relationships.

**CME AUTHOR**

Charles P. Vega, MD, Associate Professor and Residency Director, Department of Family Medicine, University of California, Irvine. Disclosure: Charles P. Vega, MD, has disclosed no relevant financial relationships.

**AUTHORS AND CREDENTIALS**

Disclosures: Katrina E. Donahue, MD, MPH; Maihan B. Vu, DrPH, MPH; Jacqueline R. Halladay, MD, MPH; Cassandra Miller, MPH; Beverly A. Garcia, MPH; Doyle M. Cummings, PharmD; Crystal W. Cene, MD, MPH; Alan Hinderliter, MD; Edwin Little, MD; and Marjorie Rachide, FNP have disclosed no relevant financial relationships.

Darren DeWalt, MD, MPH, has disclosed the following relevant financial relationships: Served as an advisor or consultant for: Merck.

Affiliations: Katrina E. Donahue, Maihan B. Vu, Jacqueline R. Halladay Cassandra Miller, Beverly A. Garcia, Crystal W. Cene, Alan Hinderliter, Darren DeWalt, University of North Carolina, Chapel Hill, North Carolina; Doyle M. Cummings, East Carolina University, Greenville, North Carolina; Edwin Little, Marjorie Rachide, primary care practices in eastern North Carolina.

## Introduction

Hypertension affects more than 68 million US adults and contributes to the excess mortality from heart disease, stroke, and chronic kidney disease (1). Despite health risks, fewer than half the people with hypertension have controlled blood pressure (1). A host of factors including medication nonadherence (2,3), unhealthy lifestyles (4), competing priorities, and provider inertia (5) contribute to suboptimal blood pressure control. In an effort to improve blood pressure control, quality improvement programs focus on patient, provider, and system changes (6). Implementing team-based approaches where providers and staff take an active role in patient care is one strategy to improve blood pressure control (7,8).

Blood pressure control initiatives that incorporate community-based participatory research (CBPR) approaches have been effective (9–11). CBPR involves collaboration among research investigators and community members to shape health improvement interventions (12). This approach is well suited to tailoring interventions to the needs and characteristics of unique health care settings. Ideally, investigators and community partners come together to investigate primary issues for blood pressure control and create effective and sustainable interventions (13). In this study, conducted from 2010 through 2012, we included health care providers, practice staff, patients, and university researchers in a CBPR process to assess patient- and practice-level barriers to blood pressure control and gather their ideas on how team-based care can enhance blood pressure control efforts. We aimed to use feedback from these stakeholders to encourage new practice behaviors and improve communication between providers and patients. The objective of this study was to assess patient and practice perceptions of the value of team-based strategies for controlling blood pressure.

## Methods

### Overview

This study was part of a 5-year cardiovascular health intervention in a county and surrounding area in the heart of the stroke belt in rural North Carolina, where stroke mortality rates are 150% of the national average (14). Key practice providers were involved in conceptualizing research design, modifying study protocol via monthly design team calls and quarterly practice meetings, and disseminating data analysis results. A community advisory board representing the project’s broad array of public health, medical, business, policy, and faith-based organizations met quarterly with the research team to offer guidance and ensure that research efforts were meeting the needs of and were sensitive to the culture of the community. Semistructured individual interviews with patients, providers, and practice staff assessed patient- and practice-level facilitators and barriers to achieving optimal blood pressure control. Interviewees were asked to use such insights to help researchers develop a blood pressure management intervention with the practices. The University of North Carolina Biomedical Institutional Review Board reviewed and approved this study.

### Participants

Eligible participants were English-speaking patients aged 18 years or older) diagnosed and managed with hypertension who were attending 1 of 5 primary care practices in rural eastern North Carolina. Six to 10 patients per practice were eligible. Hypertension was defined as systolic blood pressure greater than140 mm Hg or diastolic blood pressure greater than 90 mm Hg (15). Patients were purposely sampled to include diversity of race, sex, literacy (measured by Rapid Estimate of Adult Literacy in Medicine [REALM]) (16) and blood pressure control (controlled or not controlled as defined by their provider).

All area primary care practices (n = 8) in the study area were invited to join a collaborative effort to improve blood pressure control; 5 agreed to participate. Reasons for declining participation were lack of time or resources. To complement patient perspectives, providers and staff of each practice participated in an in-depth interview. The interviews within each practice were conducted in 2 groups; 1 with practice leadership (management, lead nurses, providers) and the other with practice staff (front office personnel, billing staff, and other clinical staff) to maximize diversity of perspectives and minimize single-source bias.

### Interviews

Face-to-face semistructured interviews explored domains of overall health concerns, barriers to optimal blood pressure control, and perceptions of improving primary care delivery for hypertensive patients (Appendix). We asked opinions about the use of care delivery teams and provided examples of how team-based care has been used effectively in other settings (17–19) to stimulate discussions on how this approach could be incorporated into the intervention. The Chronic Care Model and current evidence about team-based care was used to inform the general intervention outline (20). Trained community members conducted interviews and attempted to match interviewer with interviewee by race, which occurred 90.2% of the time. Interviews lasted approximately 60 minutes and were audio-taped and transcribed. We gave each patient a $30 monetary incentive and gave practices a $1,000 stipend to assist with the interviewing process.

### Analysis

Interview transcripts were imported into ATLAS.ti 6.2 (Scientific Software Development, GmbH, Berlin, Germany), and the study team created a codebook with operational definitions based on interview guide questions. Inductive coding techniques (21) and the constant comparison method were used (22). Two investigators reached consensus on codes and definitions, setting a priori rules for coding salient themes. The 2 coders then independently read and coded each transcript. Discrepancies were resolved through discussions of coding decisions among study team members. After reviewing all coded transcripts, the study team identified prominent themes within and across practices.

## Results

Our final sample included interviews with 41 patients and 31 practice staff (Table 1). Providers interviewed included physicians and nurse practitioners; office staff included nurses, licensed practical nurses, certified medical assistants, and front office personnel. We identified prominent themes in 3 categories: 1) perceptions of barriers to achieving controlled blood pressure, 2) perceptions of the role of practices in controlling blood pressure, and 3) perceptions of the value of team-based approaches for hypertension care (Table 2).

**Table 1:** Characteristics of Participants (N = 41)^a^, Study of Patient and Practice Perspectives on Strategies for Controlling Blood Pressure, North Carolina, 2010–2012

Characteristic	
**Age, mean (SD), y**	56.4 (11.5)
**Female**	58.5%
**Race**	
African American	68.5%
White	39.0%
American Indian	7.3%
Mixed	7.3%
**Education**	
High school or less	47.5%
**Literacy^b^**	
6th grade and below	12.2%
7th–8th grade	43.9%
High school	43.9%
**Blood pressure status**	
Taking 4 or more blood pressure medications	19.5%
Uncontrolled blood pressure^c^	57.9%

a Patient participants were drawn from 5 practices consisting of 9 key providers (physicians and nurse practitioners) and 22 office staff members (certified medical assistants, registered nurses, licensed practical nurses, and front office staff).

b Literacy assessed by using the Rapid Estimate of Adult Literacy in Medicine (16).

c Defined by provider.

**Table 2:** Key Themes Identified by Patient and Practice Staff on Barriers, Practice Role in Controlling Blood Pressure, and Team-Based Approaches for Hypertension Care, by Data Source

Themes	Patient Interviews	Practice Staff Interviews
**Patient barriers to controlling blood pressure**		
Taking prescribed medication	X	X
Dietary habits	X	X
Beliefs about hypertension	—	X
Fatalism	—	X
**Practice’s role in controlling blood pressure**		
Regular visits and check-ups	X	X
Medication compliance	X	X
Direct patient–provider communication	X	—
Media strategies (television, billboards, YouTube)	—	X
Support groups	—	X
Personal testimonies	—	X
**Team-based approaches for hypertension care**		
Value addition of contact by practice between visits	X	—
Concern about communication challenges	X	—
Concern about control and liability	—	X

Abbreviations: X, identified theme; —, did not identify theme.

### Barriers to blood pressure control

#### Medication and adherence to diet

Of the 41 patients who participated in the interviews, 41% reported their blood pressure was at the right level less than half the time. When patients were asked, “What makes it hard to keep your blood pressure under control?”, 2 main barriers were identified. First, patients said it was challenging to take medications regularly. Approximately 36% said they had missed a pill at least once in the past week. For some, remembering to take medications as scheduled was difficult. Some patients could not afford their medications. Others mentioned that they were “tired” of having to take medicine. This was influenced by the number and frequency of pills. As one patient said, “You know, what’s hard . . . to me really is sometimes I get tired [of taking medicine]. And I do know that I do need to take it. And sometimes I might not take it every day at the same time.” (Female patient, age 59, uncontrolled blood pressure)

A second barrier for patients centered on their diet. They said that an unhealthy diet that used too much salt and overeating affected blood pressure levels. Patients appeared to recognize the link between nutrition and blood pressure, but they admitted that making behavior changes was challenging. For example, as one patient stated, “Sometimes you might overeat something you know you’re not supposed to be eating like fried foods. Drinking a little bit too much, and too much salt. Food got enough salt in it already. Gotta learn how to eat it with other seasoning.” (Male patient, age 59, uncontrolled blood pressure)

Both provider and practice staff interviews showed agreement that patients’ inadequate adherence with medications affected their blood pressure. Providers recognized that patients did not take their medications because of cost or that they skipped doses to save money:

Well, I’ll ask [them] directly about it. Are you taking [them] everyday? Are you taking [them] twice a day like you’re supposed to? . . . For example, I had a patient say, ‘I take the morning stuff but I may skip a dose.’ By the same token, I’ve also had patients say, ‘Yeah, I’ve been taking it every day,’ but a 1-month prescription lasts 3 months. You know no one gave [them] a refill so what happened there?” (Practice staff)

#### Patient beliefs about hypertension

There was consensus among patients, providers, and office staff that inadequate medication adherence and poor eating habits were important barriers to blood pressure control. However, from the provider and staff perspective, the larger barrier was patients’ lack of understanding of the importance of hypertension control. Practice staff reported that some patients held certain beliefs that may affect their treatment adherence. If the patients felt well, they did not make controlling their blood pressure as a high priority. As one provider explained, “I think patients really focus on symptoms, and when they don’t have symptoms, they are not sick. . . . If they feel very well with a blood pressure of 180 over 100, it’s very hard for me to sell the blood pressure medication, especially if it’s expensive and they had to pay for it. So I wish that high blood pressure would cause pain, because then patients would be buying.” (Provider)

#### Patient fatalism

Another key barrier mentioned by providers and practice staff was patients’ belief that little could be done to prevent hypertension and its consequences. Providers and staff noted that some patients considered hypertension a problem that “runs in the family” or is “genetic” and were convinced that high blood pressure was inevitable. Practice staff shared, “We ask them their family history. They give their history and say ‘I’m sure I’m gonna have it too.’ They do think that, no matter what they do, they’re gonna get it cause their momma had it, their daddy had it, their grandma had it.” (Practice staff)

### The role of practices in controlling blood pressure

Patients identified several strategies that doctors or nurses could use to help them control blood pressure. At the top of the list were regular office visits. They valued the periodic checkups during which providers could make adjustments in prescribed medications. For example, 1 patient explained how providers could help: “The nurse practitioner at (the practice) saw a need to change it, and I’ve always wanted like a diuretic for the fluid because I feel like that would help me. . . . She saw the need to add that right in with my blood pressure medicine. That’s good if a doctor would monitor (my medications), see the need if it’s not working or you need to change, and change you over on that.” (Female patient, age 60, uncontrolled blood pressure)

Patients saw an important role for providers with antihypertensive medication management. They felt their doctors or nurses could help with medication adherence by prescribing medications that were “correct” for high blood pressure or “suited each individual.” Accessibility of refills and cost were also important factors related to adherence.

### Communication and education

A common theme that emerged from patient and provider interviews was the value of communication. Patients said it was important to have good communication with their doctors and nurses to keep their blood pressure under control. They wanted their providers to ask about how medications were working, to talk about possible side effects, and to give them information on managing their blood pressure. Providers echoed the importance of communicating with patients and observed that it was largely their job to “educate, educate, educate.” When providers and staff were asked “Where else should your patients get this message?” they discussed additional external communication strategies to reach patients. Providers mentioned the potential use of television, radio, and other media outlets given the credibility of these sources in the eyes of patients. One provider said that for his patients, “whatever comes on TV is believable.”

“TV is where [patients] see a lot of the things that they’ll mention when they come in, even medications that they want to take. When they come in, they’ll say, ‘I saw this on TV. That’s something that would help me.’ They can’t read, but they can at least see TV, and they hear what it’s telling them so TV is a good resource for some that are undereducated and can’t read . . . for those that can’t [read], TV is probably where they get most of their information.” (Provider)

Support groups and testimonies from individuals with high blood pressure were additional communication strategies that were deemed beneficial in influencing behavior change. Both practice and patient interviews showed agreement that although practices play an important role in blood pressure control, ultimately treatment success was up to the patient: “The doctor can prescribe or do whatever he needs to do. But if the patient’s not willing to go along with the program, then it’s not gonna do any good, regardless of what the doctor do.” (Male patient, Age 51, blood pressure status unknown)

### Team-based approaches for hypertension care

During the interview, patients were given an example of team-based care (Box). Patients valued verbal encouragement, calls from the doctor’s office, and the opportunity to ask questions. However, they were concerned about communication challenges if too many people were involved in their care. They feared being too far removed from their providers when given a description of team based care: “It’s got too many people included. All those people active in checking your blood pressure. I think that should just be strictly between the doctor and the patient.” (Male patient, age 59, uncontrolled blood pressure)

**Box. Components of Team-Based Care**

**Table:** 

**Patient**
Receives and learns how to use blood pressure monitor
Learns what to do when blood pressure is too high or low
Records and tells practice blood pressure readings after each med change
Receives telephone calls from the practice or health coach asking about understanding of blood pressure and ability to obtain and take medications and blood pressure readings
**Practice**
Staff members take an active role in their blood pressure care
Encourages patient to take medications and asks about side effects
Responds quickly if patient has medication side effects or the blood pressure is out of range

For some practices, the explicit use of teams in care delivery was a new concept. Providers and staff were accustomed to the physicians providing most of the patient care, although some practices were involving nurses more in a team-like approach, although not as a formal process of care delivery. “I take time to do it myself, but I may ask my nurse to sit down with a patient and talk to him about medicine or some other thing or give them literature. Or call them and see how they’re doing and emphasize the particular point of their treatment.” [Provider]

Staff members also echoed these sentiments and noted some involvement between visits, specifically when a provider wanted to make a change in a patient’s medication regimen. “Sometimes [providers will] give us a message to send to the patient, and we’ll contact them that way. Like if there’s a change to the medication, we’d be the ones normally to talk to them.” [Practice staff]

However some providers felt uneasy about giving up control and allowing other staff members to be involved in the use of a medication algorithm. “I’m comfortable with a nurse being involved in discussions with patients about medication changes that may be necessary to enhance a patient’s blood pressure control, but I would want to sign it (the new prescription) before it goes out . . . because I’m also liable . . . or without being in the communication but I would sign it before it goes out.”(Provider)

## Discussion

We conducted structured interviews of patients and practice staff in 5 primary care practices to determine key components of an effective hypertension management intervention. Important factors in improving blood pressure control included adherence to medication, dietary habits, hypertension beliefs, and regular office visits. Patients may already understand much of what is required to control blood pressure but personal, community, and health system factors limit their ability to make sustained behavior changes. In this context, new strategies that are sensitive to both patient and community needs are needed. Our study suggests team-based care approaches that enhance efforts at improving medication adherence, lifestyle management, and communication with their provider could address patients’ concerns about blood pressure control.

Team-based care models are being implemented across the country (23–25), and evidence in favor of this approach is strong (26,27). However, shifting to team-based care is not easy (28). The National Demonstration Project, which examined medical home transformation, a team-based model, indicated that patient experience did not improve in the first 2 years (29). Our study notes that patients and practices perceive some risks in the team-based model of health care delivery. Patients were receptive to the idea of team-based care but desired assurance that the providers were in charge of their care and available for direct patient–provider communications. Practice staff members were open to team-based care but not accustomed to thinking of this model as a formal approach to care. Providers also varied in their comfort with using team-based care, particularly as it relates to potential liability. Thus, movements toward team structures may need to include stakeholder education and gradual implementation as stakeholders’ values are aligned.

CBPR is an innovative approach to tailoring office-based interventions to improve hypertension care. This process engages both practices and community members in creating unique strategies to improve health care delivery that may be evidence-informed but uniquely tailored to local context. CBPR more often than not considers community-based or faith-based organizations as “the community” and as key stakeholders; health care providers are usually not considered the main community stakeholders in CBPR (30,31). Our study led to meaningful practice changes in hypertension care delivery among practices in rural North Carolina. One such change is the more purposeful use of care teams in delivery support for all of the practices’ patients with hypertension. Additionally, patients with hypertension in our study cohort also now have a community-based “phone coach” on their care team.

Findings from the CBPR approach have encouraged our research team to focus on how team-based care is implemented. For example, we created a standardized template for care delivery that allows practices to incorporate team strategies at each patient visit for hypertension care. We helped practices implement team approaches by providing on-site coaching and by gradually adding team care elements to care processes once previous activities have been vetted.

Our study has several limitations. The study was limited to a sample of patients and primary care practices in a traditional rural community where team-based care is a new concept. Additionally, selection bias was a risk, because many patient participants were identified by the practices. However, the benefit of purposeful sampling allowed us to obtain information-rich cases. Finally, it is too early to tell whether using CBPR has resulted in improved care and better blood pressure control.

 Patients and practices in our study were open to exploring the challenges to blood pressure control and to considering multiple evidence-based approaches for improving hypertension management, including the use of team-based care. When developing a team-based approach to hypertension treatment, CBPR is a useful strategy to engage patients and practices in work to ensure that interventions are informed by local context. It is hoped that locally informed intervention design will then pave the way for sustained care delivery processes long after the support from research investigators and funds has ended.

## Figures and Tables

**Table:** 

**1. The activity supported the learning objectives.**				
**Strongly Disagree**				**Strongly Agree**
1	2	3	4	5
**2. The material was organized clearly for learning to occur.**				
**Strongly Disagree**				**Strongly Agree**
1	2	3	4	5
**3. The content learned from this activity will impact my practice.**				
**Strongly Disagree**				**Strongly Agree**
1	2	3	4	5
**4. The activity was presented objectively and free of commercial bias.**				
**Strongly Disagree**				**Strongly Agree**
1	2	3	4	5

## Appendix. Patient and Practice Staff In-Depth Interview Guide Questions Related to Barriers, Practice Role for Controlling Blood Pressure, and Team Based Approaches for Hypertension Care, by Interview Topic and Data Source

**Interview Topic: Barriers to achieving controlled blood pressure**

Do you consider your blood pressure at the right level? (Patients)

From this list, what would you say is the MOST important to you to control high blood pressure? (Patients)

From this list, what would you say is the LEAST important to you to control high blood pressure? (Patients)

What makes it hard to keep your blood pressure under control (or at the right level)? (Patients)

How many times in the past week do you think you have missed a pill? How many times in the past month? (Patients)

Think about your patients who have a more difficult time keeping their blood pressure under control. Do you think they view the seriousness of hypertension differently than other patients? (Practice Staff)

Do you think some patients hold certain beliefs about hypertension that may make affect how they adhere to your treatment recommendations? (Practice Staff)

Do you think some of your patients feel that there is nothing they can do to prevent downstream effect of hypertension? (prompt: concept of fatalism) (Practice Staff)

**Interview Topic: The role of practices in controlling blood pressure**

What is the best way for your doctor or nurse to help you keep your blood pressure at the right level? (Patients)

If you have a question about a high blood pressure medicine that your doctor or nurse gives you, what would you do? (Patients)

If you need to tell your doctor something, what would you do? (Patients)

If your doctor or provider needs to get information to you, how do they reach you? (Patients)

What do you think could be done to help patients better understand the advantages of keeping their blood pressure under control? Think outside the box. What could be done in the practice? Where else should your patients get this message? (Practice Staff)

What do you think could help raise awareness about the seriousness of hypertension with your patients? (Practice Staff)

What strategies could your practice use to help patients better understand how to take their mediations as recommended? (Practice Staff)

**Interview Topic: The value of team-based approaches for hypertension care**

Based on what you’ve just heard, what you do think about this program? (Patients)

Do you think it would work? Tell me more about why that may work. (Patients)

What do you like best about what you heard? (Patients)

What are potential problems? Tell me more about the reasons they may not work. (Patients)

Do you think a team-based system like what I have described could work here? (Practice Staff)

[If practice has a team approach in place] How does this team-based system compared with what you already have in place? (Practice Staff)

[If no current structure for team-based care] What barriers do you foresee to creating a team-based system? (Practice Staff)

What do we need to keep in mind if we develop a team-based approach similar to what I described? (Practice Staff)

What kinds of team-based strategies do you think may help providers to be able to more intensively manage medications so that more patients reach goal blood pressure levels? (Practice Staff)

## Post-Test Information

To obtain credit, you should first read the journal article. After reading the article, you should be able to answer the following, related, multiple-choice questions. To complete the questions (with a minimum 70% passing score) and earn continuing medical education (CME) credit, please go to http://www.medscape.org/journal/pcd. Credit cannot be obtained for tests completed on paper, although you may use the worksheet below to keep a record of your answers. You must be a registered user on Medscape.org. If you are not registered on Medscape.org, please click on the "Register" link on the right hand side of the website to register. Only one answer is correct for each question. Once you successfully answer all post-test questions you will be able to view and/or print your certificate. For questions regarding the content of this activity, contact the accredited provider, CME@medscape.net. For technical assistance, contact CME@webmd.net. American Medical Association's Physician's Recognition Award (AMA PRA) credits are accepted in the US as evidence of participation in CME activities. For further information on this award, please refer to http://www.ama-assn.org/ama/pub/about-ama/awards/ama-physicians-recognition-award.page. The AMA has determined that physicians not licensed in the US who participate in this CME activity are eligible for **AMA PRA Category 1 Credits™**. Through agreements that the AMA has made with agencies in some countries, AMA PRA credit may be acceptable as evidence of participation in CME activities. If you are not licensed in the US, please complete the questions online, print the AMA PRA CME credit certificate and present it to your national medical association for review.

## Post-Test Questions

### Article Title: Patient and Practice Perspectives on Strategies for Controlling Blood Pressure, North Carolina, 2010–2012

**CME Questions**

1. You have identified improved control of hypertension as a quality improvement goal for your practice. In the current study by Donahue and colleagues, what were the ***main*** barriers cited by patients in improving their blood pressure control?
  - Taking medications regularly and maintaining a healthy diet
  - Getting physician appointments and a lack of exercise
  - Lack of education on hypertension and poor communication with their physician’s office
  - Getting physician appointments and too many medications
2. What was the ***most*** significant factor associated with poor blood pressure control from the perspective of providers and staff?
  - Not taking medications regularly
  - Poor diet
  - Patients’ failure to recognize the importance of hypertension
  - Poor communication with their office
3. What was the ***most*** common choice among patients as to how healthcare professionals could help them control their blood pressure?
  - Regular office visits
  - Fewer medications
  - Peer education classes
  - Interventions by nurses and dieticians
4. Which of the following statements regarding patient and healthcare provider attitudes toward team-based care in the current study is ***most*** accurate?
  - Patients were concerned about a separation between them and their provider
  - Patients did not want to receive extra calls from the provider's office
  - Providers felt that team-based care would limit their liability
  - Patients did not express a desire to ask questions

**Evaluation**

## References

[1] Centers for Disease Control and Prevention. CDC vital signs: prevalence, treatment, and control of hypertension — United States, 1999–2002 and 2005–2008. MMWR Morb Mortal Wkly Rep 2011;60(4):103–8. 21293325

[2] Bosworth HB , Dudley T , Olsen MK , Voils CI , Powers B , Goldstein MK , Racial differences in blood pressure control: potential explanatory factors. Am J Med 2006;119(1):70 e9–15. 10.1016/j.amjmed.2005.08.019 16431192

[3] Morris AB , Li J , Kroenke K , Bruner-England TE , Young JM , Murray MD . Factors associated with drug adherence and blood pressure control in patients with hypertension. Pharmacotherapy 2006;26(4):483–92. 10.1592/phco.26.4.483 16553506

[4] Ham OK , Yang SJ . Lifestyle factors associated with blood pressure control among those taking antihypertensive medication. Asia Pac J Public Health 2011;23(4):485–95. 10.1177/1010539509347941 19825840

[5] O’Connor PJ . Overcome clinical inertia to control systolic blood pressure. Arch Intern Med 2003;163(22):2677–8. 10.1001/archinte.163.22.2677 14662620

[6] Web-based educational effort for CHF patients boosts outcomes while cutting costs. Dis Manag Advis 2001;7(6):92-6, 81. 11434241

[7] Davis TC , Fredrickson DD , Arnold C , Murphy PW , Herbst M , Bocchini JA . A polio immunization pamphlet with increased appeal and simplified language does not improve comprehension to an acceptable level. Patient Educ Couns 1998;33(1):25–37. 10.1016/S0738-3991(97)00053-0 9481346

[8] O’Malley AS , Peikes D , Ginsburg PB . Making medical homes work: moving from concept to practice. Washington (DC): Center for Studying Health System Change; 2008.

[9] Allen JK , Dennison-Himmelfarb CR , Szanton SL , Bone L , Hill MN , Levine DM , Community Outreach and Cardiovascular Health (COACH) Trial: a randomized, controlled trial of nurse practitioner/community health worker cardiovascular disease risk reduction in urban community health centers. Circ Cardiovasc Qual Outcomes 2011;4(6):595–602. 10.1161/CIRCOUTCOMES.111.961573 21953407 PMC3218795

[10] Ogedegbe GO , Boutin-Foster C , Wells MT , Allegrante JP , Isen AM , Jobe JB , A randomized controlled trial of positive-affect intervention and medication adherence in hypertensive African Americans. Arch Intern Med 2012;172(4):322–6. 10.1001/archinternmed.2011.1307 22269592 PMC4669680

[11] Victor RG , Ravenell JE , Freeman A , Leonard D , Bhat DG , Shafiq M , Effectiveness of a barber-based intervention for improving hypertension control in black men: the BARBER-1 study: a cluster randomized trial. Arch Intern Med 2011;171(4):342–50. 10.1001/archinternmed.2010.390 20975012 PMC3365537

[12] Institute for Healthcare Improvement. www.ihi.org. Accessed May 1, 2012.

[13] Viswanathan M , Ammerman A , Eng E , Garlehner G , Lohr KN , Griffith D , Community-based participatory research: assessing the evidence. Evid Rep Technol Assess (Summ) 2004;(99):1–8. 15460504 PMC4780908

[14] The Heart Healthy Lenoir Project. http://www.hearthealthylenoir.com/. Accessed April 1, 2013.

[15] Chobanian AV , Bakris GL , Black HR , Cushman WC , Green LA , Izzo JL Jr , The seventh report of the Joint National Committee on Prevention, Detection, Evaluation, and Treatment of High Blood Pressure: the JNC 7 report. JAMA 2003;289(19):2560–72. 10.1001/jama.289.19.2560 12748199

[16] Murphy PW , Davis TC , Decker BC , Jackson RH . Rapid estimate of adult literacy in medicine: using a novel reading recognition test. J Read 1993;37(2):124–30.

[17] Walsh J , McDonald KM , Shojania KG , Sundaram V , Nayak S , Davies S , Closing the quality gap: a critical analysis of quality improvement strategies. Technical Review 9. (Prepared by the Stanford University-UCSF Evidence-Based Practice Center, under Contract no. 290-02-0017.) AHRQ Publication No 04-0051-3. Rockville (MD): Agency for Healthcare Research and Quality; 2005. 20734527

[18] Schroeder K , Fahey T , Ebrahim S . Interventions for improving adherence to treatment in patients with high blood pressure in ambulatory settings. Cochrane Database Syst Rev 2004;(2):CD004804. 15106262 10.1002/14651858.CD004804 PMC9036187

[19] Glynn LG , Murphy AW . Smith SM, Schroeder K, Fahey T. Interventions used to improve control of blood pressure in patients with hypertension. Cochrane Database Syst Rev 2010;( 3):CD005182. 20238338 10.1002/14651858.CD005182.pub4 PMC13248079

[20] Wagner EH , Austin BT , Davis C , Hindmarsh M , Schaefer J , Bonomi A . Improving chronic illness care: translating evidence into action. Health Aff (Millwood) 2001;20(6):64–78. 10.1377/hlthaff.20.6.64 11816692

[21] Strauss A , Corbin J . Basics of qualitative research: grounded theory procedures and techniques. Newbury Park (CA): Sage Publications; 1990.

[22] Miles M , Huberman A . Qualitative data analysis. 2nd ed. Thousand Oaks (CA): Sage; 1994.

[23] Institute for Healthcare Improvement. The breakthrough series: IHI's collaborative model for achieving breakthrough improvement. Cambridge (MA): Institute for HealthCare Improvement; 2003.

[24] Margolis PA . Designing a large-scale multilevel improvement initiative: the Improving Performance in Practice program. J Contin Educ Health Prof 2010;30(3):187–96. 10.1002/chp.20080 20872774

[25] Wise CG . Journey toward a patient-centered medical home: readiness for change in primary care practices readiness for change in primary care practices. Milbank Q 2011;89(3):399–424. 10.1111/j.1468-0009.2011.00634.x 21933274 PMC3214716

[26] Margolius D , Wong J , Goldman ML , Rouse-Iniguez J , Bodenheimer T . Delegating responsibility from clinicians to nonprofessional personnel: the example of hypertension control. J Am Board Fam Med 2012;25(2):209–15. 10.3122/jabfm.2012.02.100279 22403202

[27] Goldberg DG , Beeson T , Kuzel AJ , Love LE , Carver MC . Team-based care: a critical element of primary care practice transformation. Popul Health Manag 2013; 16(3):150–6. 10.1089/pop.2012.0059 23405875

[28] Nutting PA , Miller WL , Crabtree BF , Jaen CR , Stewart EE , Stange KC . Initial lessons from the first national demonstration project on practice transformation to a patient-centered medical home. Ann Fam Med 2009;7(3):254–60. 10.1370/afm.1002 19433844 PMC2682981

[29] Jaen CR , Ferrer RL , Miller WL , Palmer RF , Wood R , Davila M , Patient outcomes at 26 months in the patient-centered medical home National Demonstration Project. Ann Fam Med 2010;8 Suppl 1:S57–67; S92. 10.1370/afm.1121 PMC2885729 20530395

[30] Fagnan LJ , Davis M , Deyo RA , Werner JJ , Stange KC . Linking practice-based research networks and clinical and translational science awards: new opportunities for community engagement by academic health centers. Acad Med 2010;85(3):476–83. 10.1097/ACM.0b013e3181cd2ed3 20182121 PMC4059036

[31] Tapp H , Dulin M . The science of primary health-care improvement: potential and use of community-based participatory research by practice-based research networks for translation of research into practice. Exp Biol Med (Maywood) 2010;235(3):290–9. 10.1258/ebm.2009.009265 20404046

